# Subgingival microbiome at different levels of cognition

**DOI:** 10.1080/20002297.2023.2178765

**Published:** 2023-02-19

**Authors:** Nele Fogelholm, Jaakko Leskelä, Muhammed Manzoor, Jacob Holmer, Susanna Paju, Kaija Hiltunen, Hanna-Maria Roitto, Riitta Kt Saarela, Kaisu Pitkälä, Maria Eriksdotter, Kåre Buhlin, Pirkko J Pussinen, Päivi Mäntylä

**Affiliations:** a Department of Oral and Maxillofacial Diseases, University of Helsinki, Helsinki, Finland; b Division of Periodontology, Department of Dental Medicine, Karolinska Institutet, Huddinge, Sweden; c Department of Neurosciences, University of Helsinki, Helsinki, Finland; d Internal medicine and rehabilitation, Division of Geriatrics, Helsinki University Hospital Helsinki, Finland; e Population health unit, Finnish Institute for Health and Welfare, Helsinki, Finland; f Department of Social Services and Health Care, Oral Health Care, Helsinki, Finland; g Department of General Practice and Primary Health Care, University of Helsinki, Helsinki, Finland; h Unit of Primary Health Care, Helsinki University Hospital, Helsinki, Finland; i Division of Clinical Geriatrics, Department of Neurobiology, Care Sciences and Society, Karolinska Institutet, Huddinge, Sweden; j Theme Inflammation and Aging, Karolinska University Hospital, Huddinge, Sweden; k School of Medicine, Institute of Dentistry, University of Eastern Finland, Kuopio, Finland; l Oral and Maxillofacial Diseases, Kuopio University Hospital, Kuopio, Finland

**Keywords:** Oral health, cognition, cognitive decline, mini mental state examination (MMSE), subgingival microbiome, older adults

## Abstract

Oral health and declining cognition may have a bi-directional association. We characterized the subgingival microbiota composition of subjects from normal cognition to severe cognitive decline in two cohorts.

Memory and Periodontitis (MINOPAR) include 202 home-living participants (50–80 years) in Sweden. Finnish Oral Health Studies in Older Adults (FINORAL) include 174 participants (≥65 years) living in long-term care in Finland. We performed oral examination and assessed the cognitive level with Mini Mental State Examination (MMSE). We sequenced the 16S-rRNA gene (V3-V4 regions) to analyse the subgingival bacterial compositions.

The microbial diversities only tended to differ between the MMSE categories, and the strongest determinants were increased probing pocket depth (PPD) and presence of caries. However, abundances of 101 taxa were associated with the MMSE score. After adjusting for age, sex, medications, PPD, and caries, only eight taxa retained the significance in the meta-analyses of the two cohorts. Especially *Lachnospiraceae* [XIV] at the family, genus, and species level increased with decreasing MMSE.

Cognitive decline is associated with obvious changes in the composition of the oral microbiota. Impaired cognition is accompanied with poor oral health status and the appearance of major taxa of the gut microbiota in the oral cavity. Good oral health-care practices require special deliberations among older adults.

## Introduction

Cognitive impairment is a transitional condition between healthy cognition and dementia. Even though dementia predominantly affects older people, it is not a part of normal aging. Both infection and inflammation are suggested to play a role in cognitive impairment and dementia [1,2]. It has been hypothesized that long-term priming of brain glia due to bacterial entry from the host’s dysbiotic microbiota elsewhere in the body provide a slow inflammatory damage in the central nervous system [3–9].

Although experimental and clinical studies suggest an interconnection between oral dysbiotic microbiota related to common oral diseases, such as caries and periodontal diseases, and brain health/cognitive function, robust evidence is still lacking [10,11]. The oral microbiome comprises many organisms, which can induce inflammatory conditions in vulnerable individuals, such as older adults and immunocompromised people. Those inflammatory conditions can develop in several extra-oral tissues, including the brain, causing infection-induced neuroinflammation and gradual cognitive decline. In an animal study, neurodegenerative features in brain tissue, including fewer number of intact neuronal cells, were evident after repeated oral application of *Porphyromonas gingivalis*/gingipain, which suggests a role of oral pathogen in the development of neuropathology [12]. *P. gingivalis* and/or its bacterial components have been identified in post-mortem brain specimens from Alzheimer disease (AD) patients suggesting the evasion of oral pathogens to the brain [13,14]. Also, *Treponema denticola* has been identified from brain samples by molecular and immunological techniques [15], and serum antibody IgG levels to *Fusobacterium nucleatum, Prevotella intermedia* [16] and *Actinomyces naeslundii* [17] have been associated with declining cognition. Another proposed mechanism of oral-brain connection is via the communication of systemic inflammation to the brain induced by bacteraemia [18].

Although older adults with declining cognition have limited capability to maintain oral hygiene and often develop oral health problems, epidemiological studies have suggested a bi-directional association between oral health and declining cognition/dementia [19,20]. This bidirectional relationship between some neurodegenerative diseases and periodontitis is associated with an increase in inflammatory biomarkers, in IgG related to periodontopathogenic bacteria, and in periodontitis severity [21]. Mild cognitive impairment (MCI) may progress in some people to dementia, but others may remain stable or recover full function [22]. It has been argued based on experimental animal studies that brain infection is an early event much before cognitive decline and diagnosis of dementia [13]. It seems plausible that the oral dysbiosis promoting systemic inflammation may play a role in several of these neurodegenerative diseases [23].

The Mini-Mental State Examination (MMSE) [24] is the best-known and the most often used short screening tool for providing an overall measure of cognitive impairment, and it is commonly used as part of the evaluation for possible dementia [25]. Because better understanding of the origin and mechanisms of the interaction between oral microbial burden and cognitive impairment is needed, we analysed in this study the periodontal microbiome in two cohorts comprising individuals categorised by MMSE scores from normal cognition to severely weakened cognition. We investigated whether the oral (periodontal) microbiome of participants divided based on the MMSE display special features associating with cognitive decline.

## Materials and methods

### Study design and population

This study is based on two separate cohorts, which were investigated separately. Both include oral examination data and a determination of cognition performed with the MMSE test. Periodontal microbiome samples were collected similarly and analysed using the same method, in the same laboratory.

Memory and Periodontitis (MINOPAR) study. *N* = 202 home living 50–80 years old participants who were enrolled from the Karolinska Memory Clinic at the Karolinska University Hospital in Huddinge, Sweden, and from the population register in Huddinge, Sweden, comprising participants with AD, MCI, subjective cognitive impairment, and those who had no signs/symptoms of memory disorder (healthy controls). The study was conducted from 2013 to 2017. The study design has been published by Holmer et al. [26] including precise description of diagnostic criteria, control population criteria, and study exclusion criteria. Ethical approval for the study was obtained from the Regional Ethical Review Board in Stockholm (2012/652–31/1).

Finnish Oral Health Studies in Older Adults (FINORAL) study. *N* = 174 65 years or older residents in the capital area of Helsinki, Finland, who are living in long-term care (nursing homes and assisted living facilities). This cohort comprises the dentate participants of the FINORAL, which is a random subsample of participants of the nutrition study that included older residents in the capital area of Helsinki [27]. Study was conducted from 2017 to 2019. The study design has been earlier published by Hiltunen et al. [28] and Julkunen et al. [29]. The City of Helsinki and the Ethics Committee of the Hospital District of Helsinki and Uusimaa approved the study protocol (HUS/2042/2016 and HUS/968/2017).

Both cohorts adhere to the guidelines of the Declaration of Helsinki. Written informed consent was obtained from all participants and/or their proxies.

### Data collection

All study participants either completed a questionnaire about personal data (medical, dental, educational information etc.) (MINOPAR), or a registered nurse most familiar with the participants in each facility filled in a questionnaire concerning study participants’ demographic characteristics (age, sex, education) (FINORAL).

In both cohorts, the clinical oral examination (conducted for MINOPAR at the Department of Dental Medicine, Karolinska Institutet, Huddinge, Sweden; for FINORAL at each participant’s long-term care facility) comprised a comprehensive assessment of the oral soft and hard tissues including a periodontal examination comprising probing pocket depth (PPD), both at six sites on all existing teeth, and registration of caries lesions [26,29]. In MINOPAR, the MMSE examination was performed prior to the oral examination at the same visit; other diagnoses were obtained from medical records, and use of medications was self-reported (questionnaire). In FINORAL, the MMSE examination was performed in connection with the implementation of the nutrition study, and the residents’ medical diagnoses and use of medications were retrieved from medical records.

After completion of the oral examination, the deepest or the most representative periodontal pocket was selected in each quadrant for subgingival microbial sampling. In MINOPAR, the quadrant to be sampled was isolated with cotton rolls/pads and a saliva ejector. Supragingival plaque was carefully removed at the selected sampling site using a sterile curette, leaving the subgingival biofilm undisturbed. All samples were taken with a new individual sterile curette with a single pull, in a coronal direction, from the base of the periodontal pocket. The procedure was repeated in all four quadrants. For FINORAL, due to the exceptional conditions, there are some exceptions to the sampling protocol: no saliva ejector was available, cotton rolls/pads were used, if possible, not all supragingival plaque could be removed from everyone, all samples were taken with one sterile curette. The procedure was repeated in all four quadrants where teeth were present. The samples were pooled in 1.5 mL microcentrifuge tubes (Thermo Fisher Scientific®) containing PCR grade water (Roche®) and stored at−80°C until further processing.

### Sample processing and microbiota profiling

The methodology of DNA extraction, PCR amplification and 16S rRNA gene amplicon sequencing is detailed elsewhere [30,31]. In brief, DNA was extracted from the subgingival samples using FastDNATM Spin Kit for Soil (MP Biomedicals) according to the manufacturer’s instructions. PCR amplifications were performed on ARKTIK Thermal Cyclers (Finnzymes/Thermo Scientific) and the V3–V4 hypervariable regions of the 16S rRNA gene were amplified with primers reported previously [32] using Illumina MiSeq (Illumina Inc., San Diego, CA, United States; paired-end sequencing; read lengths: forward: 326 bp; reverse: 278 bp) at the DNA Sequencing and Genomics Laboratory of the Institute of Biotechnology, University of Helsinki, Finland.

Primers were trimmed from paired-end read with cutadapt (v. 2.8 with Python 3.8.10) [33] and were merged together to reconstruct full-length sequences following the standard operating procedure (SOP) for MiSeq data using mothur pipeline (v. 1.45.2) [34]. The following criteria were applied: (i) removal of homopolymers (>8 bp), (ii) removal of sequence reads containing ambiguous bases, and (iii) removal of assembled reads>435 bp in length. This reduced the number of assembled reads, ensuring high quality of data. Sequences with chimeric reads were removed by UCHIME algorithm incorporated in the mothur. We used Silva reference database (v. 132) [35] for alignment and Human Oral Microbiome Database (HOMD v. 15.1) [36] for taxonomy and identified the Operational Taxonomic Unit (OTU). Singleton sequences were removed from data during the mothur analysis. The raw data are available in the European Nucleotide Archive (accession numbers: PRJEB35923 for MINOPAR and PRJEB57333 for FINORAL).

### Statistical analyses

For statistical analyses, both cohorts were stratified according to points achieved in MMSE test: normal cognition or very mild cognitive decline (hereafter referred to as normal cognition; 25–30 points), mild cognitive decline (20–24 points), moderate cognitive decline (10–19 points), and severe cognitive decline (0–9).

The categorical variables were described as numbers and percentages (%), and the continuous variables as means and standard deviations (SDs) or medians with interquartile range (IQR, shown as 25th and 75th percentiles).

We removed OTUs with fewer than six sequence reads, samples with a low number of sequence reads (<10000 reads), and a high number of sequences (> = 150000 reads) before analysis. After these steps, the final number of good-quality sequences was 17,193 729 (mean ± SD: 78870 ± 23 155 per sample) and 10,921 316 (mean ± SD: 43685 ± 9 526 per sample) for MINOPAR and FINORAL, respectively.

We compared the alpha diversity measures (observed richness and Shannon index) between the MMSE categories using Phyloseq (v.1.34.0). Kruskal–Wallis tests, Kendall rank correlation, Jonckheere-Terpstra test, and linear regression (combined model with multiple variables) were used for diversity analyses. Results were shown in two models; model 1, adjusted for age and sex, and model 2, adjusted for age, sex, education, caries, smoking, medications, and PPD.

The differences in microbial community composition in terms of beta diversity between the MMSE categories were tested using Bray-Curtis dissimilarities with the permutational multivariate analysis of variance (PERMANOVA) in R package vegan (v. 2.6–0) and visualization with non-metric multidimensional scaling (NMDS). Differentially abundant OTUs at different taxonomy levels (focusing on OTUs, genera, and species that had more than one read in more than one-fourth of the samples) were identified using general linear models with a negative binomial distribution using the R package DESeq2 (1.30.1).

The relative abundance of the 18 most common genera was compared in both cohorts. We tested the associations between the differential abundant taxa and MMSE scores in crude model. Finally, for the taxa that were significant in crude model of either cohort, we performed meta-analyses with additional adjustments for age, sex, number of medications, PPD≥4 mm and presence of caries.

The statistical analyses were performed in the R version 4.0.5, and we set the level of statistical significance to *p* < 0.05.

## Results

The characteristics of the participants of MINOPAR (*n* = 202) and FINORAL (*n* = 174) are presented in Table 1.

**Table 1.** Characteristics of the participants.

			MMSE category (score)					
	Cohort	Subgroup	Normal^1^ (25–30)	Mild (20–24)	Moderate (10–19)	Severe (0–9)	Total	*p*-value^2^
n (%)	MINOPAR		176 (87.1)	19 (9.4)	7 (3.5)	0	202 (53.7)	
	FINORAL		13 (7.5)	25 (14.4)	93 (53.4)	43 (24.7)	174 (46.3)	
	Total		189 (50.2)	44 (11.7)	100 (26.6)	43 (11.4)	376 (100)	
Age years mean (SD)	MINOPAR		67.3 (7.0)	70.8 (8.0)	70.4 (5.4)	0 (0)	67.7 (7.1)	**0.021**
	FINORAL		84.8 (6.6)	82.8 (9.8)	83.8 (8.7)	81.4 (7.5)	83.0 (8.4)	0.270
	Total		68.5 (8.2)	77 (10.5)	82.9 (9.1)	81.4 (7.5)	74.8 (10.9)	**<0.001**
Female n (%)	MINOPAR		95 (54.0)	11 (57.9)	2 (28.6)	0 (0)	108 (53.5)	0.614
	FINORAL		11 (84.6)	16 (64.0)	63 (67.7)	36 (83.7)	126 (72.4)	0.222
	Total		106 (56.1)	27 (61.4)	65 (65.0)	36 (83.7)	234 (62.2)	**0.002**
Education n (%)	MINOPAR	1–12 years	104 (59.1)	14 (73.7)	6 (85.7)	0 (0)	124 (61.4)	0.082
		University	72 (40.9)	5 (26.3)	1 (14.3)	0 (0)	78 (38.6)	
	FINORAL	1–12 years	10 (83.3)	18 (75)	74 (88)	36 (87.8)	138 (79.3)	0.266
		University	2 (16.7)	6 (25)	10 (12)	5 (12.2)	23 (13.2)	
Type of residence n (%)	MINOPAR	At home	176 (100)	19 (100)	7 (100)	0 (0)	202 (100)	
	FINORAL	Nursing home	6 (46.2)	11 (44.0)	44 (47.3)	19 (44.2)	80 (46.0)	
		Assisted living	7 (53.8)	14 (56.0)	49 (52.7)	24 (55.8)	94 (54.0)	
Smoking n (%)	MINOPAR	Current	10 (5.7)	3 (15.8)	1 (14.2)	0 (0)	14 (6.9)	0.915
		Earlier	79 (44.9)	5 (26.3)	3 (42.9)	0 (0)	87 (43.1)	
		Never	87 (49.4)	11 (57.9)	3 (42.9)	0 (0)	101 (50.0)	
	FINORAL	Current	0 (0)	0 (0)	0 (0)	0 (0)	0 (0)	**0.001**
		Earlier	7 (70)	7 (50.0)	17 (30.4)	4 (28.6)	35 (37.2)	
		Never	3 (30)	7 (50.0)	39 (69.6)	10 (71.4)	59 (62.8)	
Partner status n (%)	MINOPAR	Together^3^	121 (68.8)	14 (73.7)	6 (85.7)	0 (0)	141 (69.8)	0.407
		Single^4^	55 (31.2)	5 (26.3)	1 (14.3)	0 (0)	61 (30.2)	
	FINORAL	Together^3^	1 (7.7)	7 (28.0)	19 (20.7)	11 (25.6)	38 (22.0)	0.526
		Single^4^	12 (92.3)	18 (72.0)	73 (79.3)	32 (74.4)	135 (78.0)	
Medications mean (SD)	MINOPAR		2.5 (2.7)	3.0 (3.9)	4.4 (2.3)	0 (0)	2.6 (2.9)	0.187
	FINORAL		9.3 (5.3)	10.6 (3.5)	9.3 (3.4)	8.0 (2.9)	9.2 (3.6)	**0.013**
BMI, kg/m^2^ mean (SD)	MINOPAR		25.8 (4.0)	24.8 (4.5)	25.6 (3.1)	0 (0)	25.7 (4)	0.354
	FINORAL		25.2 (3.7)	27.9 (7.0)	26.4 (4.9)	25.7 (5.6)	26.3 (5.3)	0.542
Diabetes n (%)	MINOPAR		18 (10.3)	2 (10.5)	2 (28.6)	0 (0)	22 (10.9)	0.368
	FINORAL		5 (38.5)	8 (32.0)	15 (16.1)	8 (18.6)	36 (20.7)	0.056
	Total		23 (12.2)	10 (22.7)	17 (17)	8 (4.9)	58 (15.4)	0.150
N of teeth med (IQR)	MINOPAR		26 (24–28)	26 (22.5–28)	25 (17–27)	0 (0)	26 (24–28)	0.365
	FINORAL		14 (6–20)	14 (9–21)	13 (6–19)	13 (6–20)	13 (6–20)	0.623
	Total		26 (23–28)	22 (13–26)	13 (6–20.25)	13 (6–20)	23 (13–27)	**<0.001**
Teeth with increased PPD med (IQR)	MINOPAR	4–5 mm	5 (2–7)	5 (2.5–9)	3 (2–6)	0 (0)	5 (2–7)	0.680
		≥6 mm	0 (0–1)	2 (0–2)	2 (0.5–3)	0 (0)	0 (0–2)	**0.011**
	FINORAL	4–5 mm	1 (0–5)	2 (0–4)	2 (0–5)	1 (0–6)	2 (0–5)	0.808
		≥6 mm	0 (0–0)	0 (0–0)	0 (0–0)	0 (0–0)	0 (0–0)	0.594
	Total	4–5 mm	5 (2–7)	4 (1–6)	2 (0–5)	1 (0–6)	4 (1–7)	**<0.001**
		≥6 mm	0 (0–1)	0 (0–2)	0 (0–1)	0 (0–0)	0 (0–1)	**0.010**
Caries present n (%)	MINOPAR		28 (15.9)	3 (15.8)	0 (0)	0 (0)	31 (15.3)	0.548
	FINORAL		7 (53.8)	21 (84.0)	62 (67.4)	29 (67.4)	119 (68.4)	0.806
	Total		35 (18.5)	24 (54.5)	62 (62.0)	29 (67.4)	150 (39.9)	**<0.001**

^1^normal/very mild cognitive decline. ^2^p-values calculated for the trend using Jonckheere-Tersptra test. The significant p-values are bolded. ^3^ In MINOPAR living together; in FINORAL married, not living together. ^4^ Widowed, single, divorced.

The mean age (SD) of MINOPAR participants was 67.7 (7.1) years, while the FINORAL participants were older, 74.8 (10.9) years. Thus, the age range in the two cohorts was from 51 to 101 years. The number of females was 108 (53.5%) and 126 (72.4%) in MINOPAR and FINORAL, respectively. The MINOPAR participants lived at home and 78 (38.6%) had academic education, while the FINORAL participants lived in nursing home [80 (46.0%)] or assisted living facilities [94 (54.0%)] and 23 (13.2%) had university level education. 101 (50.0%) and 59 (62.8%) of MINOPAR and FINORAL participants had never smoked.

The median (IQR) number of teeth was 26 (24—28) and 13 (6—20) in MINOPAR and FINORAL, and caries was observed in 31(15.3%) and 119 (68.4%) of the participants, respectively. 189 (93.6%) in MINOPAR and 54 participants (33.8%) in FINORAL had deepened periodontal pockets (at least 4 mm deep).≥6 mm periodontal pockets were found in 87 (43.1%) and 34 (21.3%) participants in MINOPAR and FINORAL, respectively.

**Figure 1.**
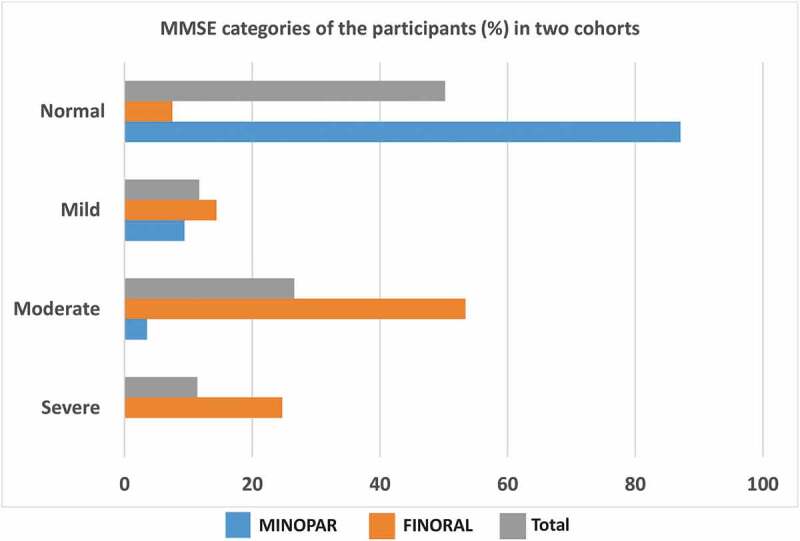
MMSE categories in MINOPAR and FINORAL. MINOPAR (*n* = 202) and FINORAL (*n* = 174) participants were divided into subgroups according to the MMSE score. The scores for normal cognition, and mild, moderate and severe decline of cognition were 25–30, 20–24, 10–19, and 0–9.

We divided the cohorts into categories based on the MMSE test results. In MINOPAR, 176 (87.1%) had normal cognition, while 19 (9.4%) and 7 (3.5%) had mild or moderate cognitive decline, respectively. In FINORAL, the corresponding frequencies were 13 (7.5%), 25 (14.4%), 93 (53.4%), respectively, and 43 (24.7%) had severe cognitive decline. Accordingly, the two cohorts comprised a continuum of MMSE results complementing each other (Figure 1).

**Figure 2.**
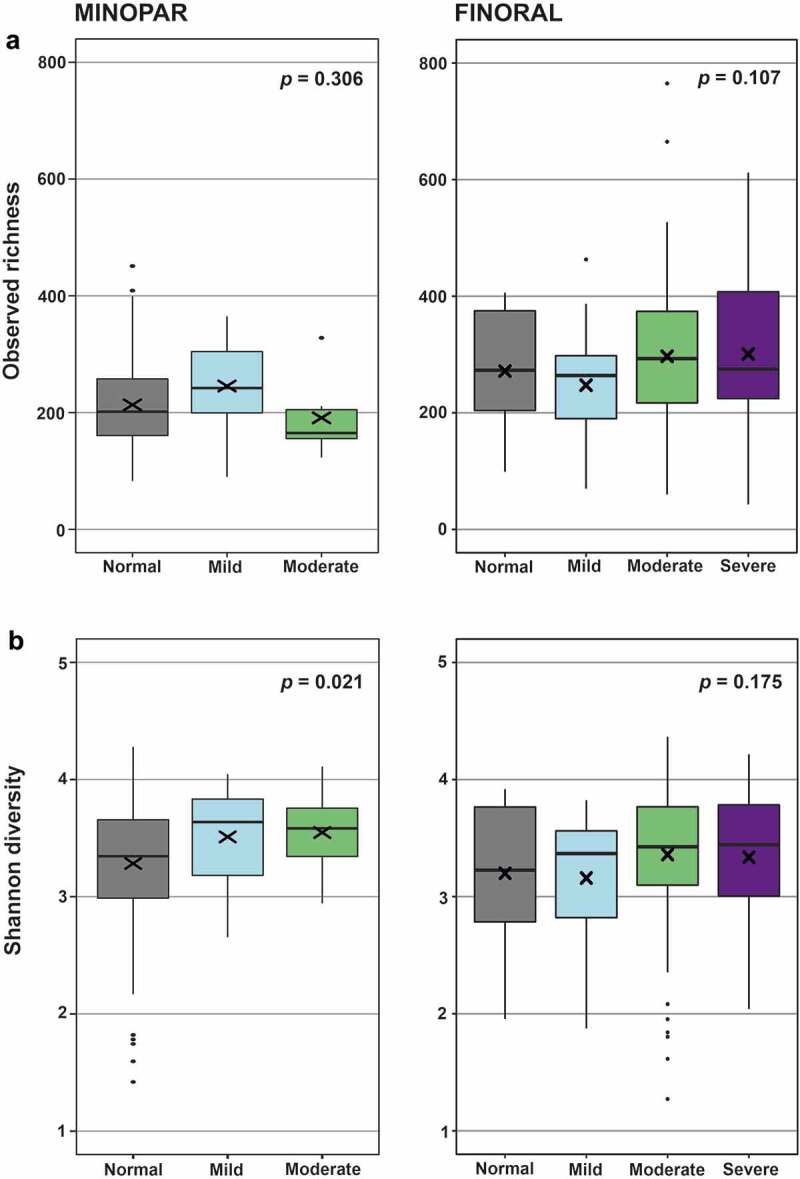
Alpha diversity of the oral microbiome in the MMSE categories. MINOPAR (*n* = 202) and FINORAL (*n* = 174) participants were divided into subgroups according to the MMSE score as normal, mild, moderate, and severe cognitive decline. Alpha diversity as observed richness and Shannon index were calculated for the subgingival microbiome composition in both cohorts. p-values for the significance of the difference between the MMSE groups are shown. The box plots present median (x), mean (line), IQR (box), and 95% CI (error bars).

We amplified and sequenced 16S rRNA gene V3-V4 hypervariable regions from subgingival plaque samples resulting in 28,115,045 good-quality sequence reads. We investigated first the richness and evenness of the microbial communities, the alpha diversity, by comparing observed richness and Shannon index between the MMSE categories (Table 2 , Figure 2). We observed a significant declining trend between MMSE categories in MINOPAR (Shannon index 0.021), whereas in FINORAL the alpha diversity did not differ between the categories. Alpha diversity measures differed significantly between sexes and participants with or without caries or increased PPD and had a significant correlation with the number of teeth (Table 2). In multivariate linear models, alpha diversity did not associate with MMSE categories, but gender, number of teeth, caries and PPD were significantly associated in either of the cohorts (Table 2).

**Table 2.** Comparisons of alpha diversity according to the characteristics of the cohorts.

		MINOPAR				FINORAL			
		Observed richness		Shannon index		Observed richness		Shannon index	
		*p*-value^1^, univariate	Beta (SE), *p*-value^2^	*p*-value^1^, univariate	Beta (SE), *p*-value^2^	*p*-value^1^, univariate	Beta (SE), *p*-value^2^	*p*-value^1^, univariate	Beta (SE), *p*-value^2^
MMSE category	Mild	0.306	18.4 (15.9), 0.249	**0.021**	0.248 (0.121), 0.062	0.107	−18.9 (40.2), 0.637	0.175	0.002 (0.203), 0.993
	Moderate		−26.2 (25.1), 0.306		0.317 (0.194), 0.106		34.6 (34.6), 0.320		0.206 (0.175), 0.242
	Severe		-		-		42.1 (37.6), 0.265		0.168 (0.190), 0.377
Age (years)		0.919	0.828 (0.701), 0.243	0.566	0.003 (0.005), 0.498	0.363	0.317 (1.23), 0.800	0.132	−0.005 (0.006), 0.431
Sex (male/female)		**0.001**	**-30.3 (9.21), 0.001**	**0.012**	**-0.175 (0.071), 0.014**	0.218	30.1 (21.3), 0.160	0.80	0.192 (0.108), 0.077
Education (lower/higher)		0.803		0.096		0.668		0.733	
Smoking (never/ever)		0.585		0.152		0.084		0.163	
Medications (n)		0.942		0.286		0.403		0.827	
Teeth (n)		0.808	0.001 (1.03), 0.999	0.162	**0.017 (0.008), 0.037**	**<0.001**	**4.42 (1.37), 0.002**	**<0.001**	0.010 (0.006), 0.159
Caries (no/yes)		**0.014**	20.7 (12.9), 0.111	**0.020**	**0.196 (0.099), 0.048**	0.084	−9.32 (20.5), 0.651	0.261	−0.100 (0.104), 0.342
Teeth with increased PPD		**<0.001**	**6.27 (0.924), <0.001**	0.449	−0.004 (0.007), 0.537	**0.048**	0.634 (1.96), 0.747	0.094	0.007 (0.010), 0.500

^1^Jonckheere-Terpstra for MMSE categories, Kruskall-Wallis for categorical variables (sex, education, smoking, caries) and Kendall correlation analyses (age, medications, teeth, PPD) for continuous variables; ^2^ Linear regression analyses for the association of MMSE with observed richness and Shannon index adjusted for age and covariates with statistically significant p-values in univariate analyses. The significant p-values are bolded.

In univariate analysis, differences were observed in the beta-diversity between the MMSE categories, but they did not reach statistical significance (MINOPAR, *p* = 0.163; FINORAL, *p* = 0.077). Beta-diversity was significantly different between sexes, and between participants with or without caries or increased PPD and correlated with age and number of medications in use (Table 3 , Figure S1). In multivariate analyses, increased PPD had the strongest association with beta-diversity in MINOPAR explaining almost 4% of the variability, while the beta-diversity in FINORAL was associated with the presence of caries, which explained 1.2% of the variability.

**Table 3.** Comparisons of beta diversity according to the characteristics of the cohorts.

	MINOPAR		FINORAL	
	Beta diversity		Beta diversity	
	*p*-value^1^, univariate	R-squared, *p*-value^2^	*p*-value^1^, univariate	R-squared, *p*-value^2^
MMSE category	0.163	0.012, 0.110	0.077	0.021, 0.275
Age (years)	**0.041**	**0.009, 0.028**	**0.013**	**0.011, 0.013**
Sex (male/female)	0.059	0.007, 0.104	**0.031**	0.007, 0.352
Education (lower/higher)	0.165		0.368	
Smoking (never/ever)	0.601		0.366	
Medications (n)	0.070	0.006, 0.164	**0.008**	**0.010, 0.038**
Teeth (n)	0.065	0.007, 0.110	0.094	0.009, 0.066
Caries (no/yes)	**0.050**	0.005, 0.328	**0.021**	**0.012, 0.016**
Teeth with increased PPD	**<0.001**	**0.039, <0.001**	**0.036**	0.007, 0.334

^1^Kruskall-Wallis for categorical variables (MMSE, sex, education, smoking, caries) and Kendall correlation analyses (age, medications, teeth, PPD) for continuous variables; ^2^ Linear regression analyses for the association of MMSE with beta diversity adjusted for age and covariates with statistically significant p-values in univariate analyses. The significant p-values are bolded.

Subgingival microbiota was mainly composed of five genera: *Fusobacterium*, *Prevotella*, *Streptococcus*, *Veillonella*, and *Capnocytophaga*. The 18 most abundant genera are presented according to the MMSE categories in Supplemental Table S1. *Streptococcus* (*p* = 0.010) and *Actinomyces* (*p* = 0.041) exhibited a significant decreasing, and *Tannerella* (*p* = 0.013), *Treponema* (*p* = 0.003), *Corynebacterium* (*p* = 0.009), and Saccharibacteria G-5 (*p* = 0.003) a significant increasing trend with declining MMSE score in FINORAL cohort. In MINOPAR, no significant trends were observed.

**Figure 3.**
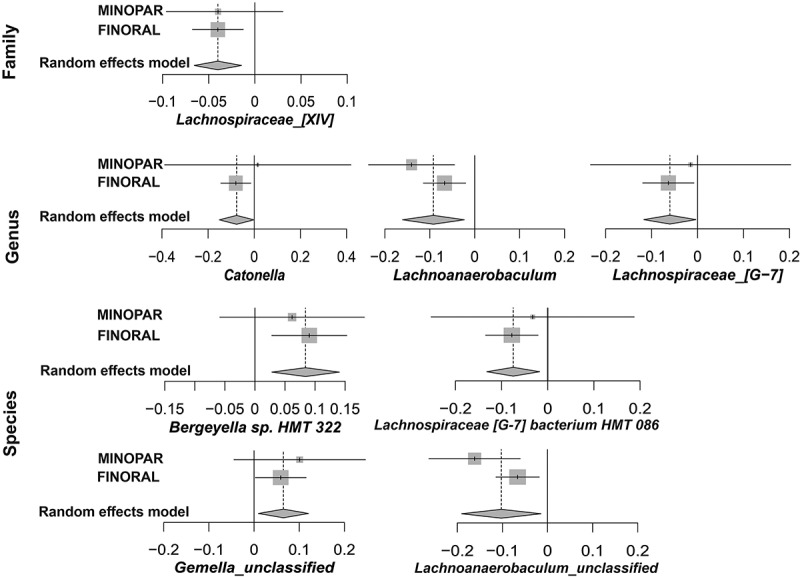
Meta-analyses of the significant OTUs. the forest plots display the results of the final random effects models produced by meta-analyses of all significant taxa of either cohort, adjusted for age, sex, number of medications, PPD≥6 mm and presence of caries. Effect size and 95% CI are shown separately for MINOPAR, FINORAL, and their meta-analyses.

The differential abundance of several OTUs associated significantly with the MMSE score in general linear models (Supplemental Table S2). In MINOPAR, the number of associated taxa was 39, while in FINORAL 67 taxa were associated with MMSE score. The largest estimates were observed for *Prevotella saccharolytica* (−0.585/MMSE unit) and *Saccharibacteria* [G-1] *bacterium* (−0.437/MMSE unit) in MINOPAR, and *Bacteroidetes* (−0.184/MMSE unit) and *Saccharibacteria* (−0.177/MMSE unit) families in FINORAL. All significant taxa were further subjected to meta-analyses of both cohorts with additional adjustments for age, sex, number of medications, PPD≥6 mm and presence of caries (Supplemental Table S3). In the final random effects models with full adjustment (Figure 3), *Lachnospiraceae* [XIV] family [beta (95% CI), p-value] [−0.04 (−0.07; −0.01), 0.002], genera *Lachnospiraceae* [G-7] [−0.08 (−0.15; −0.00), 0.046], *Lachnoanaerobaculum* [−0.09 (−0.16; −0.02), 0.009] and *Catonella* [−0.08 (−0.15; −0.00), 0.046], and species *Lachnospiraceae* [G-7] bacterium HMT 086 [−0.07 (−0.13; −0.02), 0.011] and one *Lachnoanaerobaculum* unclassified species [−0.10 [−0.19; −0.02), 0.022] were significantly associated with MMSE score. Additionally, *Bergeyella* sp. HMT 322 [0.08 (0.03; 0.14), 0.003] and *Gemella* unclassified [0.07 (0.009; 0.12), 0.021] displayed a significant association. The forest plots of the taxa with suggestive p-values in the random effect models are presented in Supplemental Figure S2.

## Discussion

We showed among 376 older adults, that cognitive decline is associated with substantial compositional changes of the subgingival microbiota. Although the microbial richness or evenness did not differ significantly between the MMSE categories, we found 101 different taxa that were associated with cognitive decline. When the obvious differences between the two cohorts were considered, especially the abundance of the *Lachnospiraceae* [XIV] family members increased with declining cognition independently of residence type, age, sex, medications, and presence of periodontitis or caries. Genus *Gemella* and *Bergeyella* sp. were depleted in participants with cognitive impairment. Thus, the number of associated taxa decreased markedly after adjusting for the presence of deepened periodontal pockets or caries: Oral health status was the strongest determinant of the subgingival microbiota composition.

The two cohorts of the study represent very different segments of older adults with respect to age, type of housing, level of education and state of cognition. FINORAL cohort comprised Finnish long-term care facility residents from the capital area with markedly impaired ability to function. They were significantly older than MINOPAR cohort comprising Swedish residents also from the greater capital area but living independently at home. We assessed MMSE scores for both cohorts: FINORAL participants categorised as having normal cognition or mild cognitive decline formed a minority (22%), while MINOPAR comprised mainly such participants (96.5%) and none of them had severe cognitive impairment. However, these differences offer a perspective to both a wide age range and variation in the level of cognition. Strengths of the study include the careful evaluations of oral health status and registering of putative confounders, such as smoking, education level, BMI, and diabetes. The differences between the two cohorts may also be regarded as a limitation. Also, other important risk factors for cognitive decline, such as nutrition or cholesterol levels were not recorded.

The most important prerequisite for comparing these cohorts was that the microbiota was analysed in the same laboratory with a similar 16S rRNA gene amplicon sequencing method. Cohorts were examined separately in all statistical analyses; data were not combined at any stage. The main objective of the study, to recognise special features of the subgingival microbiota in groups divided based on MMSE and to identify possible marker species, was carried out by conducting a meta-analysis with microbial taxa selected among those displaying significant log2 fold changes in linear models. With this approach, we were able to avoid the challenges caused by several dissimilarities of the two data sets, and to produce an insight of microbial taxa that associate with the decrease of MMSE considering confounding factors.

We observed a declining trend of alpha diversity (Shannon index) between MINOPAR MMSE categories but not in FINORAL. In multivariate models, this association was lost. However, in these models, alpha diversity was associated significantly with gender, caries, and PPD in MINOPAR, and with the number of teeth in both cohorts. MINOPAR participants had a higher number of teeth and teeth with increased PPD, while FINORAL participants had fewer teeth and a lower number of teeth with increased PPD. In MINOPAR, the strongest association of beta-diversity was with increased PPD explaining almost 4% of the variability. In FINORAL, on the other hand, beta-diversity was associated with the presence of caries which explained 1.2% of the variability. Caries was very common in FINORAL (68% of all participants) while in MINOPAR only 15% had caries teeth. The oldest old population living in long-term facilities has often lost teeth with the most severe periodontitis earlier in life but suffer often from root caries and milder forms of periodontitis [37].

In a Danish study, participants with MMSE scores less than 24 had significantly more both coronal and root surface caries than those with higher score [38]. Caries experience of institutionalized older adults has been associated with dementia, disability and lack of oral care [39], but less is known about the role of caries in brain health. Periodontitis has been hypothesized as being one of the most common potential risk factors for the cognitive impairment and development of dementia/neurodegenerative diseases. Our earlier study suggested that marginal periodontitis is associated with both early cognitive impairment and AD [26]. In a retrospective study, 10-year exposure to periodontitis increased significantly the risk of developing dementia (AD) (odds ratio, OR 1.7) [40]. A recent meta-analysis concluded that periodontitis is associated with cognitive impairment [41], and subjects with moderate or severe periodontitis were at 2.13 times greater risk of developing dementia compared with persons without moderate or severe periodontitis [42]. One earlier study linked MMSE and oral health: in a 5-year prospective study, severe periodontitis and periodontal inflammation were associated with increased odds for MCI among older adults segmented by a chemosensory test and cognitive scores by MMSE [43]. Controversies also exist, since in our large cohort study conducted in Sweden deep periodontal probing was not associated with incidence of dementia [44]. Importantly, however, in a large Japanese study, having had periodontal treatment was associated with a lower risk for dementia [45].

There is some evidence that in individuals with cognitive impairment or AD, the subgingival microbiota exhibits a shift typical of periodontitis [30] but that the microbial signatures change to favor opportunistic species with increasing AD severity [46]. Periodontal pathogens, such as *Porphyromonas gingivalis* and *Treponema denticola*, have been found in post-mortem brain tissue samples of AD patients [13–15] suggesting a direct bacterial translocation into the brain, which is also supported by the animal studies [47]. Among studies using targeted methods, elevated serum antibodies to periodontal disease bacteria were associated with future cognitive impairment, AD patients compared to healthy, and risk for developing incident AD [16,17,48,49].

It is possible that weakening cognition (determined by MMSE) and consequent deficient oral hygiene will result that certain bacterium achieve a dominant role in periodontal biofilm. Oral hygiene is known to be poor especially among vulnerable, care-dependent institutionalized older people [50,51] [52], particularly among those who need assistance with oral hygiene [53,54], and fast deterioration of oral health because of caries or periodontal problems is possible [55]. In our earlier study [29] oral disease burden was associated with functional and cognitive decline according to MMSE among long-term care facility residents in line with prior studies [56,57].

In the present study, the relative abundance of only a few genera correlated significantly with the MMSE categories: *Streptococcus* and *Actinomyces* displayed a decreasing trend, while *Tannerella*, *Treponema*, *Corynebacterium*, and *Saccharibacteria* [G-5] presented an increasing trend with declining cognition. Relative abundances of several common oral bacterial genera were significantly different for FINORAL and MINOPAR cohorts. Higher abundancies of periodontitis-related genera, *Tannerella*, *Treponema*, and *Porphyromonas* in MINOPAR can be explained by the higher number of teeth with increased PPD, especially PPD≥6 mm. *Fusobacterium*, more abundant in MINOPAR, and *Prevotella*, more abundant in FINORAL, both genera also common in periodontal diseases, are not traditionally considered as pathogenic for periodontitis as bacteria in genera *Tannerella*, *Treponema* and *Porphyromonas* [58]. *Streptococcus* were twice as abundant in MINOPAR as in FINORAL, though caries was more common in FINORAL. In older long-term care living populations especially root caries is common [59], and it does not need streptococci to develop [60]. In FINORAL higher abundance of *Veillonella*, common bacteria in the gastro-intestinal tract including oral mucous membrane, can be explained by the inadequate oral hygiene of long-term care residents, as previously published in the FINORAL population [29,61].

We found associations between 101 taxa and MMSE score in univariate analyses. When the confounders were considered and the two cohorts subjected to a meta-analysis, eight taxa retained the significance. Among them was *Lachnospiraceae* [XIV] family, which belongs to the phylum of *Firmicutes*, the class of *Clostridia* and includes several genera, such as *Catonella*, *Lacnoanaerobaculum*, *Lachnospiraceae* [G-7], and *Oribacterium*. The family is anaerobic and among the most abundant taxa in the human gut microbiota [62]. Several studies have shown that gut *Lachnospiraceae* [XIV] is less abundant in Alzheimer’s disease compared to healthy subjects [63,64] and associated with a better performance on MMSE and other cognitive assessments [42,65]. The advantageous properties are believed to derive from *Lachnospiraceae*’s ability to ferment indigestible carbohydrate producing beneficial metabolites [66]. The health-promoting metabolites, such as butyrate, are crucial for proper function of the intestinal barrier thus maintaining immune homeostasis, preventing translocation of endotoxins, and promoting anti-inflammatory properties [62]. On the other hand, *Lachnospiraceae* may associate with inflammation, metabolic syndrome, liver diseases, and chronic kidney diseases [62]. The present study, however, confirms and strengthen our earlier results that increased subgingival *Lachnospiraceae* is associated with low MMSE score and Alzheimer’s disease [30]. The association was independent from residence type, age, sex, medications, and presence of periodontitis or caries. Nevertheless, the relative abundance of subgingival *Lachnospiraceae* was less than 1% while it may be predominant in the gut with a 40% abundance [64]. The result suggests that species highly common in the gut appear in the oral cavity in dementia. *Lachnoaerobaculum* has been detected in the oral biofilm among all adults despite the caries status [67]) but it has also been associated with smoking [68]. Thus, whether an altered microbiota in the oral cavity is a cause or consequence and how it relates to the *Lachnospiraceae* taxa in the gut remains to be investigated.

After carefully considering demographic factors and oral health status, we did not observe significant differences in the diversity of the microbiota between the MMSE categories. However, we show that cognitive decline is associated with obvious changes in the composition of the subgingival microbiota and most of them associate with poor oral health. Considering the putative contribution of oral health to dementia, good oral health-care practices require special deliberations at all ages and at all stages of cognition.

## Supplementary Material

Supplemental Material
