# Supplementary material for: Subgingival microbiome at different levels of cognition
Source: J Oral Microbiol. 2023 Feb 19;15(1):2178765. doi: 10.1080/20002297.2023.2178765 (PMC9946326; doi:10.1080/20002297.2023.2178765)
Supplement: Supplemental Material [file ZJOM_A_2178765_SM8440.zip › Supplementary files/Supplemental_tables_and_figures_Anonymous.docx]

**Supplemental Table 1**. **Relative abundance of 18 most common bacterial genera in Cohort 1 and Cohort 2 according to the MMSE categories.**

| Genus | Cohort | Relative abundance (%), median (IQR) | | | | p-value^2^ |
| --- | --- | --- | --- | --- | --- | --- |
|  |  | **Normal^1^** | **Mild** | **Moderate** | **Severe** |  |
| *Streptococcus* | Cohort 1 | 18.0 (9.38-25.2) | 13.0 (11.1-18.9) | 11.9 (10.9-13.3) |  | 0.884 |
|  | Cohort 2 | **9.06 (5.58-12.4)** | **5.01 (3.05-13.5)** | **4.53 (2.36-9.90)** | **3.90 (1.80-8.22)** | **0.010** |
| *Prevotella* | Cohort 1 | 3.43 (1.48-5.93) | 5.62 (3.93-7.05) | 1.31 (1.11-3.52) |  | 0.284 |
|  | Cohort 2 | 8.73 (5.70-20.1) | 10.7 (6.22-17.2) | 10.7 (6.22-17.2) | 12.7 (6.12-19.2) | 0.411 |
| *Veillonella* | Cohort 1 | 0.97 (0.25-2.28) | 0.68 (0.37-2.69) | 0.69 (0.48-3.42) |  | 0.684 |
|  | Cohort 2 | 7.79 (4.04-16.7) | 9.92 (4.64-23.1) | 7.48 (3.37-16.8) | 6.17 (1.10-12.6) | 0.063 |
| *Capnocytophaga* | Cohort 1 | 3.63 (1.31-8.72) | 3.54 (2.49-8.94) | 8.40 (4.84-10.6) |  | 0.166 |
|  | Cohort 2 | 5.57 (1.68-8.77) | 2.61 (0.57-7.74) | 4.48 (1.21-10.1) | 2.20 (0.84-8.88) | 0.704 |
| *Fusobacterium* | Cohort 1 | 18.0 (9.38-25.2) | 13.0 (11.1-18.9) | 11.9 (10.9-13.3) |  | 0.306 |
|  | Cohort 2 | 4.45 (2.26-7.69) | 6.11 (0.87-8.71) | 6.24 (2.76-11.4) | 7.81 (2.18-13.6) | 0.094 |
| *Selenomonas* | Cohort 1 | 1.59 (0.61-3.62) | 2.40 (1.09-7.82) | 2.47 (1.06-3.37) |  | 0.069 |
|  | Cohort 2 | 3.62 (1.57-6.17) | 2.59 (0.92-4.26) | 4.62 (2.76-7.66) | 4.16 (1.67-5.74) | 0.402 |
| *Campylobacter* | Cohort 1 | 4.07 (2.52-5.84) | 3.85 (2.82-6.56) | 3.06 (2.95-4.54) |  | 0.785 |
|  | Cohort 2 | 3.19 (2.01-5.07) | 3.89 (2.83-4.77) | 4.39 (2.65-7.10) | 3.80 (1.71-6.01) | 0.892 |
| *Actinomyces* | Cohort 1 | 2.13 (0.87-4.26) | 2.07 (0.52-4.04) | 5.10 (1.58-5.94) |  | 0.970 |
|  | Cohort 2 | **2.34 (1.80-3.50)** | **2.47 (1.39-4.46)** | **2.00 (0.99-3.99)** | **1.12 (0.66-3.54)** | **0.041** |
| *Leptotrichia* | Cohort 1 | 2.06 (0.58-3.43) | 1.38 (0.92-3.31) | 5.89 (4.68-8.07) |  | 0.816 |
|  | Cohort 2 | 1.67 (1.16-5.65) | 2.99 (0.36-9.17) | 3.45 (0.51-8.01) | 1.21 (0.10-5.12) | 0.306 |
| *Dialister* | Cohort 1 | 0.26 (0.06-0.81) | 0.71 (0.29-1.01) | 0.09 (0.02-0.37) |  | 0.303 |
|  | Cohort 2 | 0.80 (0.10-1.81) | 0.49 (0.18-1.18) | 0.50 (0.13-1.51) | 1.10 (0.30-1.66) | 0.113 |
| *Saccharibacteria* G-1 | Cohort 1 | 1.79 (0.70-3.24) | 1.27 (0.47-5.29) | 4.24 (1.88-5.57) |  | 0.276 |
|  | Cohort 2 | 0.44 (0.00-0.74) | 0.28 (0.00-1.73) | 0.78 (0.05-2.56) | 1.03 (0.09-2.49) | 0.161 |
| *Lachnoanaerobaculum* | Cohort 1 | 0.13 (0.03-0.32) | 0.14 (0.02-0.42) | 0.35 (0.25-1.57) |  | 0.058 |
|  | Cohort 2 | 0.27 (0.07-0.45) | 0.33 (0.00-0.81) | 0.38 (0.13-0.88) | 0.42 (0.08-0.76) | 0.458 |
| *Tannerella* | Cohort 1 | 1.43 (0.47-2.88) | 1.19 (0.61-3.39) | 1.82 (0.83-2.92) |  | 0.816 |
|  | Cohort 2 | **0.23 (0.02-1.28)** | **0.01 (0.00-0.19)** | **0.21 (0.01-0.70)** | **0.36 (0.08-1.03)** | **0.013** |
| *Treponema* | Cohort 1 | 3.68 (0.75-7.12) | 3.79 (1.49-8.66) | 1.91 (0.94-3.71) |  | 0.752 |
|  | Cohort 2 | **0.21 (0.02-1.65)** | **0.49 (0.05-0.96)** | **0.74 (0.09-2.05)** | **1.16 (0.22-3.53)** | **0.003** |
| *Rothia* | Cohort 1 | 1.49 (0.30-4.76) | 0.85 (0.23-2.71) | 0.53 (0.32-7.24) |  | 0.409 |
|  | Cohort 2 | 0.12 (0.05-0.51) | 0.23 (0.03-0.57) | 0.06 (0.01-0.21) | 0.05 (0.01-0.28) | 0.082 |
| *Porphyromonas* | Cohort 1 | 1.04 (0.33-4.13) | 2.30 (0.58-3.68) | 6.05 (2.19-10.3) |  | 0.091 |
|  | Cohort 2 | 0.09 (0.01-3.28) | 0.17 (0.01-1.27) | 0.38 (0.01-2.86) | 0.31 (0.01-1.98) | 0.789 |
| *Corynebacterium* | Cohort 1 | 2.25 (0.71-6.59) | 2.81 (0.94-5.34) | 8.99 (3.23-10.5) |  | 0.293 |
|  | Cohort 2 | **0.01 (0.00-0.12)** | **0.01 (0.00-0.10)** | **0.01 (0.00-0.11)** | **0.03 (0.00-0.18)** | **0.009** |
| *Saccharibacteria G-5* | Cohort 1 | 0.00 (0.21-1.51) | 0.06 (1.01-2.52) | 0.02 (0.15-0.35) |  | 0.376 |
|  | Cohort 2 | **0.00 (0.00-0.00)** | **0.00 (0.00-0.00)** | **0.00 (0.00-0.57)** | **0.00 (0.00-1.54)** | **0.003** |

^1^ normal /very mild cognitive decline. ^2^p-value for trend across the MMSE categories (Joncheere-Tersptra test). The significant p-values are bolded.

**Supplemental Table 2. Significant associations of the MMSE score with subgingival taxa in Cohort 1 and Cohort 2.**

| **Cohort** | **Level** | | | **Taxon** | **log2 fc (SE)** | **p-value** |
| --- | --- | --- | --- | --- | --- | --- |
| **Cohort 1** | Family |  |  | *Burkholderiaceae* | 0.149 (0.060) | 0.013 |
|  | Family |  |  | *Erysipelotrichaceae* | 0.172 (0.066) | 0.008 |
|  |  | Genus |  | *Solobacterium* | 0.167 (0.069) | 0.015 |
|  |  |  | Species | Otu00149 *S. moorei* | 0.156 (0.070) | 0.026 |
|  | Family |  |  | *Lachnospiraceae*_[XIV] | -0.069 (0.033) | 0.040 |
|  |  | Genus |  | *Lachnospiraceae* [G-3] | -0.172 (0.085) | 0.043 |
|  |  |  | Species | Otu00141 *Lachnospiraceae* [G-3] *bacterium* HMT 100 | -0.183 (0.086) | 0.031 |
|  |  | Genus |  | *Lachnoanaerobaculum* | -0.105 (0.047) | 0.024 |
|  |  |  | Species | Otu00080 *Lachnoanaerobaculum* unclassified | -0.129 (0.049) | 0.009 |
|  | Family |  |  | *Neisseriaceae* | 0.112 (0.044) | 0.011 |
|  |  | Genus |  | *Neisseria* | 0.140 (0.060) | 0.020 |
|  |  |  | Species | Otu00030 *Neisseria oralis* | 0.322 (0.097) | 0.001 |
|  | Family |  |  | *Propionibacteriaceae* | 0.150 (0.53) | 0.005 |
|  | Family |  |  | *Selenomonadaceae* | -0.092 (0.038) | 0.015 |
|  |  | Genus |  | *Mitsuokella* | -0.410 (0.11) | <0.001 |
|  |  |  | Species | Otu00024 *Selenomonas noxia* | -0.150 (0.051) | 0.003 |
|  |  |  | Species | Otu00242 *Selenomonas dianae* | -0.258 (0.107) | 0.016 |
|  |  | Genus |  | *Slackia* | -0.247 (0.082) | 0.003 |
|  |  |  | Species | Otu00316 *Slackia exigua* | -0.271 (0.085) | 0.002 |
|  |  | Genus |  | *Haemophilus* | 0.181 (0.062) | 0.003 |
|  |  | Genus |  | *Pseudopropionibacterium* | 0.175 (0.062) | 0.005 |
|  |  |  | Species | Otu00138 *Actinomyces* sp. HMT 525 | 0.159 (0.047) | <0.001 |
|  |  |  | Species | Otu00010 *Actinomyces* unclassified | 0.109 (0.046) | 0.018 |
|  |  |  | Species | Otu00241 *Actinomyces* sp. HMT 897 | -0.167 (0.075) | 0.028 |
|  |  |  | Species | Otu00069 *Aggregatibacter* unclassified | -0.198 (0.087) | 0.023 |
|  |  |  | Species | Otu00403 *Bacteroidales* [G-2] *bacterium* HMT 274 | 0.129 (0.065) | 0.049 |
|  |  |  | Species | Otu00036 *Capnocytophaga sputigena* | 0.237 (0.084) | 0.005 |
|  |  |  | Species | Otu00176 *Catonella* sp. HMT 451 | -0.451 (0.152) | 0.003 |
|  |  |  | Species | Otu00110 *Catonella morbi* | 0.175 (0.066) | 0.008 |
|  |  |  | Species | Otu00358 *Fusobacterium* unclassified | 0.078 (0.038) | 0.040 |
|  |  |  | Species | *Otu00108 Leptotrichia sp. HMT 219* | 0.471 (0.112) | <0.001 |
|  |  |  | Species | Otu00168 Gemella unclassified | 0.140 (0.070) | 0.045 |
|  |  |  | Species | Otu00066 *Porphyromonas pasteri* | -0.163 (0.081) | 0.045 |
|  |  |  | Species | Otu00130 *Prevotella* sp. HMT 472 | 0.254 (0.109) | 0.020 |
|  |  |  | Species | Otu00279 *Rothia mucilaginosa* | 0.179 (0.071) | 0.011 |
|  |  |  | Species | Otu00094 *Saccharibacteria* (TM7) [G-1] *bacterium* HMT 348 | -0.437 (0.142) | 0.002 |
|  |  |  | Species | Otu00043 *Saccharibacteria* (TM7) [G-1] unclassified | -0.144 (0.069) | 0.036 |
|  |  |  | Species | Otu00109 *Saccharibacteria* (TM7) [G-1] *bacterium* HMT 488 | 0.270 (0.129) | 0.037 |
|  |  |  | Species | Otu00107 *Veillonellaceae* [G-1] *bacterium* HMT 155 | -0.172 (0.070) | 0.014 |
| **Cohort 2** | Family |  |  | *Actinomycetaceae* | 0.038 (0.016) | 0.021 |
|  |  |  | Species | Otu000202 *Actinomyces massiliensis* | 0.133 (0.052) | 0.011 |
|  | Family |  |  | *Bacteroidaceae* | -0.125 (0.045) | 0.006 |
|  |  | Genus |  | *Bacteroidaceae* [G-1] | -0.107 (0.047) | 0.022 |
|  |  |  | Species | Otu000072 *B*. [G-1] *bacterium* HMT 272 | -0.138 (0.050) | 0.005 |
|  | Family |  |  | *Bacteroidetes* [F-1] | -0.183 (0.056) | 0.001 |
|  | Family |  |  | *Eggerthellaceae* | -0.098 (0.037) | 0.008 |
|  |  | Genus |  | *Cryptobacterium* | -0.110 (0.036) | 0.002 |
|  |  |  | Species | Otu000123 *C. curtum* | -0.083 (0.036) | 0.021 |
|  | Family |  |  | *Lachnospiraceae*_[XIV] | -0.045 (0.013) | <0.001 |
|  |  | Genus |  | *Lachnospiraceae*_[G-7] | -0.095 (0.027) | <0.001 |
|  |  |  | Species | Otu000220 *L. [G-7] bacterium* HMT 086 | -0.091 (0.028) | 0.001 |
|  |  | Genus |  | *Lachnoanaerobaculum* | -0.068 (0.023) | 0.003 |
|  |  |  | Species | Otu000045 *L.* unclassified | -0.068 (0.023) | 0.003 |
|  |  |  | Species | Otu000372 *L.* unclassified | -0.075 (0.028) | 0.007 |
|  |  | Genus |  | *Catonella* | -0.095 (0.031) | 0.002 |
|  |  |  | Species | Otu000167 *C. morbi* | -0.121 (0.034) | <0.001 |
|  |  | Genus |  | *Oribacterium* | -0.057 (0.019) | 0.004 |
|  |  |  | Species | Otu000064 *Oribacterium* sp. HMT 078 | -0.041 (0.020) | 0.047 |
|  | Family |  |  | *Neisseriaceae* | 0.138 (0.035) | <0.001 |
|  |  | Genus |  | *Neisseria* | 0.118 (0.042) | 0.005 |
|  |  |  | Species | Otu000041 *N. macacae* | 0.240 (0.050) | <0.001 |
|  | Family |  |  | *Pasteurellaceae* | 0.102 (0.033) | 0.024 |
|  | Family |  |  | *Peptococcaceae* | -0.107 (0.040) | 0.007 |
|  |  | Genus |  | *Peptococcus* | -0.089 (0.040) | 0.025 |
|  | Family |  |  | *Saccharibacteria* (TM7) [F-2] | -0.177 (0.059) | 0.002 |
|  |  | Genus |  | *Saccharibacteria* (TM7) [G-5] | -0.157 (0.058) | 0.007 |
|  |  |  | Species | Otu000027 *Saccharibacteria* (TM7) [G-5] *bacterium HMT 356* | -0.175 (0.059) | 0.003 |
|  |  |  | Species | Otu000069 *Saccharibacteria* (TM7) [G-1] *bacterium* HMT 349 | -0.106 (0.043) | 0.015 |
|  | Family |  |  | *Spirochaetaceae* | -0.048 (0.024) | 0.043 |
|  | Family |  |  | *Streptococcaceae* | 0.119 (0.021) | <0.001 |
|  |  | Genus |  | *Streptococcus* | 0.117 (0.021) | <0.001 |
|  |  |  | Species | Otu000268 *S. oralis subsp. dentisani* clade 058 | 0.105 (0.023) | <0.001 |
|  |  |  | Species | Otu000003 *S. oralis subsp. dentisani* clade 058 | 0.077 (0.021) | <0.001 |
|  |  |  | Species | Otu000248 *S. oralis subsp. dentisani* clade 058 | 0.069 (0.021) | 0.001 |
|  |  |  | Species | Otu000292 *S. oralis subsp. dentisani* clade 058 | 0.064 (0.021) | 0.002 |
|  |  |  | Species | Otu000359 *S. oralis* subsp. *dentisani* clade 058 | 0.056 (0.025) | 0.026 |
|  |  |  | Species | Otu000084 *S. parasanguinis* clade 411 | 0.104 (0.036) | 0.004 |
|  |  |  | Species | Otu000304 *S. anginosus* | -0.052 (0.026) | 0.045 |
|  | Family |  |  | *Synergistaceae* | -0.076 (0.034) | 0.027 |
|  |  |  | Species | Otu000131 *Fretibacterium* unclassified | -0.094 (0.041) | 0.022 |
|  |  |  | Species | Otu000177 *Fretibacterium fastidiosum* | -0.071 (0.034) | 0.040 |
|  |  | Genus |  | *Bergeyella* | 0.077 (0.029) | 0.009 |
|  |  |  | Species | Otu000124 *Bergeyella* sp. HMT 322 | 0.106 (0.031) | <0.001 |
|  |  | Genus |  | *Granulicatella* | -0.079 (0.026) | 0.029 |
|  |  | Genus |  | *Haemophilus* | 0.204 (0.034) | <0.001 |
|  |  |  | Species | *Haemophilus parainfluenza* | 0.121 (0.037) | 0.001 |
|  |  | Genus |  | *Peptostreptococcaceae* [XI][G-6] | -0.163 (0.051) | 0.001 |
|  |  | Genus |  | *Ralstonia* | 0.112 (0.042) | 0.007 |
|  |  | Genus |  | *Treponema* | -0.049 (0.024) | 0.039 |
|  |  |  | Species | Otu000090 *Treponema*_unclassified | -0.175 (0.045) | <0.001 |
|  |  |  | Species | Otu000022 *T. socranskii* | -0.061 (0.025) | 0.015 |
|  |  |  | Species | Otu000391 *Atopobium* unclassified | -0.069 (0.027) | 0.012 |
|  |  |  | Species | Otu000056 *Atopobium* unclassified | -0.059 (0.024) | 0.014 |
|  |  |  | Species | Otu000183 *Olsenella uli* | -0.070 (0.033) | 0.038 |
|  |  |  | Species | Otu000121 *Cardiobacterium valvarum* | 0.201 (0.059) | <0.001 |
|  |  |  | Species | Otu000176 *Corynebacterium durum* | 0.195 (0.043) | <0.001 |
|  |  |  | Species | Otu000062 *Granulicatella adiacens* | 0.053 (0.024) | 0.027 |
|  |  |  | Species | Otu000211 *Mogibacterium timidum* | -0.063 (0.026) | 0.017 |
|  |  |  | Species | Otu000358 *Parvimonas micra* | -0.052 (0.025) | 0.035 |
|  |  |  | Species | Otu000051 *Porphyromonas pasteri* | 0.240 (0.047) | <0.001 |
|  |  |  | Species | Otu000366 *Prevotella* unclassified | -0.079 (0.035) | 0.023 |
|  |  |  | Species | Otu000152 *Selenomonas dianae* | -0.171 (0.058) | 0.003 |
|  |  |  | Species | Otu000330 *Selenomonas* unclassified | -0.073 (0.031) | 0.017 |
|  |  |  | Species | Otu000192 *Selenomonas sputigena* | 0.081 (0.027) | 0.003 |
|  |  |  | Species | Otu000209 Veillonella_unclassified | 0.046 (0.023) | 0.042 |
|  |  |  | Species | Otu000221 Veillonella sp. HMT 780 | -0.093 (0.046) | 0.043 |

General linear models with a negative binomial distribution using the DESeq2 for MMSE score as a continuous variable.

**Supplemental Table 3. Meta-analyses of all significant taxa of Cohort 1 and Cohort 2.**

|  |  | Cohort 1 | | Cohort 2 | | RANDOM EFFECT MODEL | | | | |
| --- | --- | --- | --- | --- | --- | --- | --- | --- | --- | --- |
| Taxon | **Level** | **log2 fc (SE)** | **p-value** | **log2 fc (SE)** | **p-value** | | **log2 fc (SE)** | **p-value** | **hetero-geneity** | **hetero-geneity p-value** |
| *Actinomyces massiliensis* | Species | -0.034 (0.064) | 0.594 | 0.029 (0.122) | 0.814 | | -0.02 (0.058) | 0.728 | 0.000 | 0.647 |
| *Actinomyces sp. HMT 525* | Species | 0.118 (0.05) | **0.017** | -0.016 (0.026) | 0.534 | | 0.044 (0.064) | 0.495 | 0.826 | 0.017 |
| *Actinomyces sp. HMT 897* | Species | 0.067 (0.178) | 0.707 | 0.048 (0.05) | 0.336 | | 0.049 (0.048) | 0.304 | 0.000 | 0.917 |
| *Actinomyces_unclassified* | Species | -0.018 (0.047) | 0.708 | -0.012 (0.023) | 0.587 | | -0.013 (0.02) | 0.515 | 0.000 | 0.919 |
| *Actinomycetaceae* | Family | -0.017 (0.038) | 0.643 | 0.02 (0.016) | 0.215 | | 0.012 (0.019) | 0.542 | 0.000 | 0.359 |
| *Aggregatibacter_unclassified* | Species | -0.146 (0.327) | 0.654 | -0.029 (0.21) | 0.890 | | -0.064 (0.177) | 0.720 | 0.000 | 0.763 |
| *Atopobium_unclassified* | Species | 0.092 (0.222) | 0.677 | -0.055 (0.025) | **0.031** | | -0.048 (0.05) | 0.335 | 0.000 | 0.510 |
| *Bacteroidaceae* | Family | 0.018 (0.525) | 0.972 | -0.041 (0.149) | 0.784 | | -0.036 (0.143) | 0.800 | 0.000 | 0.914 |
| *Bacteroidaceae [G-1] bacterium HMT 272* | Species | 0.03 (0.525) | 0.955 | -0.046 (0.17) | 0.789 | | -0.038 (0.162) | 0.812 | 0.000 | 0.891 |
| *Bacteroidaceae_[G-1]* | Genus | 0.038 (0.525) | 0.943 | -0.05 (0.152) | 0.740 | | -0.044 (0.146) | 0.765 | 0.000 | 0.872 |
| *Bacteroidales [G-2] bacterium HMT 274* | Species | 0.163 (0.072) | **0.023** | -0.037 (0.184) | 0.841 | | 0.118 (0.096) | 0.221 | 0.030 | 0.310 |
| *Bacteroidetes_[F-1]* | Family | 0.093 (0.432) | 0.830 | -0.07 (0.184) | 0.702 | | -0.045 (0.171) | 0.793 | 0.000 | 0.728 |
| *Bergeyella* | Genus | 0.008 (0.056) | 0.886 | 0.07 (0.03) | **0.020** | | 0.052 (0.033) | 0.113 | 0.000 | 0.329 |
| *Bergeyella sp. HMT 322* | Species | 0.062 (0.061) | 0.314 | 0.09 (0.032) | **0.005** | | 0.084 (0.029) | **0.003** | 0.000 | 0.680 |
| *Burkholderiaceae* | Family | -0.008 (0.062) | 0.900 | 0.036 (0.039) | 0.362 | | 0.023 (0.035) | 0.507 | 0.000 | 0.553 |
| *Capnocytophaga sputigena* | Species | 0.304 (0.091) | **0.001** | 0.048 (0.21) | 0.821 | | 0.235 (0.124) | 0.057 | 0.199 | 0.264 |
| *Cardiobacterium valvarum* | Species | -0.07 (0.243) | 0.774 | 0.05 (0.199) | 0.803 | | 0.002 (0.155) | 0.992 | 0.000 | 0.704 |
| *Catonella* | Genus | 0.015 (0.206) | 0.940 | -0.08 (0.033) | **0.016** | | -0.076 (0.038) | **0.046** | 0.000 | 0.648 |
| *Catonella morbi* | Species | 0.046 (0.219) | 0.834 | -0.092 (0.035) | **0.010** | | -0.084 (0.051) | 0.100 | 0.000 | 0.535 |
| *Corynebacterium durum* | Species | -0.05 (0.303) | 0.868 | 0.226 (0.046) | **0.000** | | 0.195 (0.108) | 0.071 | 0.000 | 0.368 |
| *Erysipelotrichaceae* | Family | 0.112 (0.179) | 0.529 | 0.019 (0.024) | 0.436 | | 0.022 (0.032) | 0.506 | 0.000 | 0.603 |
| *Fretibacterium fastidiosum* | Species | 0.063 (0.077) | 0.417 | -0.08 (0.035) | **0.021** | | -0.026 (0.067) | 0.693 | 0.648 | 0.092 |
| *Fretibacterium_unclassified* | Species | 0.056 (0.258) | 0.829 | -0.104 (0.041) | **0.011** | | -0.096 (0.059) | 0.105 | 0.000 | 0.540 |
| *Gemella_unclassified* | Species | 0.101 (0.074) | 0.174 | 0.059 (0.029) | **0.040** | | 0.065 (0.028) | **0.021** | 0.000 | 0.599 |
| *Granulicatella adiacens* | Species | -0.106 (0.053) | **0.046** | 0.032 (0.081) | 0.690 | | -0.05 (0.067) | 0.457 | 0.510 | 0.153 |
| *Haemophilus* | Genus | 0.019 (0.067) | 0.776 | 0.071 (0.115) | 0.538 | | 0.032 (0.058) | 0.581 | 0.000 | 0.696 |
| *Haemophilus parainfluenzae* | Species | -0.075 (0.066) | 0.258 | 0.075 (0.117) | 0.520 | | -0.026 (0.076) | 0.734 | 0.197 | 0.264 |
| *Lachnoanaerobaculum* | Genus | -0.141 (0.049) | **0.004** | -0.067 (0.024) | **0.005** | | -0.092 (0.035) | **0.009** | 0.452 | 0.177 |
| *Lachnoanaerobaculum_unclassified* | Species | -0.162 (0.052) | **0.002** | -0.066 (0.024) | **0.007** | | -0.103 (0.045) | **0.022** | 0.640 | 0.095 |
| *Lachnospiraceae [G-3] bacterium HMT 100* | Species | -0.126 (0.235) | 0.591 | -0.016 (0.131) | 0.905 | | -0.042 (0.116) | 0.715 | 0.000 | 0.681 |
| *Lachnospiraceae [G-7] bacterium HMT 086* | Species | -0.033 (0.112) | 0.771 | -0.078 (0.029) | **0.007** | | -0.075 (0.029) | **0.011** | 0.000 | 0.697 |
| *Lachnospiraceae_[G-3]* | Genus | -0.123 (0.231) | 0.595 | -0.014 (0.122) | 0.907 | | -0.038 (0.109) | 0.726 | 0.000 | 0.677 |
| *Lachnospiraceae_[G-7]* | Genus | -0.015 (0.111) | 0.891 | -0.064 (0.028) | **0.025** | | -0.06 (0.029) | **0.038** | 0.000 | 0.673 |
| *Lachnospiraceae_[XIV]* | Family | -0.04 (0.036) | 0.266 | -0.04 (0.014) | **0.004** | | -0.04 (0.013) | **0.002** | 0.000 | 0.996 |
| *Mitsuokella* | Genus | -0.032 (0.448) | 0.943 | 0.008 (0.21) | 0.969 | | 0.001 (0.19) | 0.996 | 0.000 | 0.935 |
| *Mogibacterium timidum* | Species | 0.048 (0.173) | 0.782 | -0.056 (0.027) | **0.038** | | -0.051 (0.038) | 0.181 | 0.000 | 0.553 |
| *Neisseria* | Genus | -0.011 (0.242) | 0.964 | 0.039 (0.21) | 0.854 | | 0.017 (0.159) | 0.913 | 0.000 | 0.877 |
| *Neisseria macacae* | Species | -0.091 (0.387) | 0.815 | 0.058 (0.21) | 0.782 | | 0.024 (0.186) | 0.897 | 0.000 | 0.736 |
| *Neisseriaceae* | Family | 0.071 (0.047) | 0.130 | 0.061 (0.205) | 0.768 | | 0.07 (0.046) | 0.123 | 0.000 | 0.961 |
| *Olsenella uli* | Species | 0.072 (0.161) | 0.654 | -0.067 (0.034) | 0.053 | | -0.051 (0.057) | 0.376 | 0.000 | 0.399 |
| *Oribacterium* | Genus | -0.02 (0.156) | 0.899 | -0.031 (0.02) | 0.116 | | -0.031 (0.02) | 0.115 | 0.000 | 0.941 |
| *Oribacterium sp. HMT 078* | Species | -0.037 (0.182) | 0.840 | -0.027 (0.021) | 0.207 | | -0.027 (0.021) | 0.201 | 0.000 | 0.957 |
| *Parvimonas micra* | Species | 0.045 (0.053) | 0.390 | -0.047 (0.027) | 0.080 | | -0.012 (0.044) | 0.777 | 0.586 | 0.120 |
| *Peptococcaceae* | Family | 0.268 (0.091) | **0.003** | -0.11 (0.042) | **0.008** | | 0.07 (0.184) | 0.703 | 0.930 | 0.000 |
| *Peptococcus* | Genus | 0.248 (0.09) | **0.006** | -0.089 (0.042) | **0.035** | | 0.07 (0.163) | 0.668 | 0.913 | 0.001 |
| *Peptostreptococcaceae_[XI][G-6]* | Genus | 0.098 (0.348) | 0.780 | -0.11 (0.052) | **0.034** | | -0.101 (0.075) | 0.179 | 0.000 | 0.556 |
| *Porphyromonas pasteri* | Species | -0.121 (0.285) | 0.672 | 0.064 (0.179) | 0.719 | | 0.011 (0.156) | 0.946 | 0.000 | 0.583 |
| *Prevotella sp. HMT 472* | Species | 0.094 (0.387) | 0.809 | 0.005 (0.211) | 0.981 | | 0.025 (0.185) | 0.892 | 0.000 | 0.840 |
| *Prevotella_unclassified* | Species | 0.04 (0.253) | 0.875 | -0.076 (0.038) | **0.045** | | -0.072 (0.045) | 0.105 | 0.000 | 0.650 |
| *Propionibacteriaceae* | Family | 0.138 (0.057) | **0.015** | 0.05 (0.029) | 0.082 | | 0.08 (0.042) | 0.058 | 0.485 | 0.164 |
| *Pseudopropionibacterium* | Genus | 0.162 (0.063) | **0.011** | 0.026 (0.039) | 0.510 | | 0.084 (0.065) | 0.197 | 0.703 | 0.067 |
| *Ralstonia* | Genus | -0.049 (0.065) | 0.454 | 0.02 (0.138) | 0.884 | | -0.036 (0.06) | 0.552 | 0.000 | 0.650 |
| *Rothia mucilaginosa* | Species | 0.096 (0.076) | 0.206 | 0.018 (0.19) | 0.924 | | 0.085 (0.071) | 0.235 | 0.000 | 0.705 |
| *Saccharibacteria (TM7) [G-5] bacterium HMT 356* | Species | -0.034 (0.084) | 0.688 | -0.107 (0.21) | 0.610 | | -0.044 (0.078) | 0.576 | 0.000 | 0.745 |
| *Saccharibacteria_(TM7)_[F-2]* | Family | -0.045 (0.081) | 0.581 | -0.101 (0.21) | 0.631 | | -0.052 (0.076) | 0.493 | 0.000 | 0.804 |
| *Saccharibacteria_(TM7)_[G-1]_unclassified* | Species | -0.007 (0.263) | 0.979 | -0.041 (0.179) | 0.820 | | -0.03 (0.148) | 0.839 | 0.000 | 0.916 |
| *Saccharibacteria_(TM7)_[G-5]* | Genus | -0.066 (0.08) | 0.414 | -0.1 (0.21) | 0.635 | | -0.07 (0.075) | 0.351 | 0.000 | 0.879 |
| *Selenomonadaceae* | Family | -0.061 (0.04) | 0.134 | -0.012 (0.018) | 0.518 | | -0.025 (0.024) | 0.295 | 0.182 | 0.269 |
| *Selenomonas dianae* | Species | 0.014 (0.242) | 0.954 | -0.203 (0.063) | **0.001** | | -0.173 (0.094) | 0.067 | 0.000 | 0.386 |
| *Selenomonas noxia* | Species | -0.164 (0.054) | **0.003** | 0.049 (0.034) | 0.152 | | -0.053 (0.103) | 0.605 | 0.909 | 0.001 |
| *Selenomonas sputigena* | Species | 0.029 (0.058) | 0.620 | 0.065 (0.054) | 0.224 | | 0.048 (0.04) | 0.225 | 0.000 | 0.643 |
| *Slackia* | Genus | -0.115 (0.085) | 0.177 | -0.005 (0.023) | 0.832 | | -0.032 (0.049) | 0.520 | 0.361 | 0.211 |
| *Slackia exigua* | Species | -0.154 (0.09) | 0.088 | -0.016 (0.024) | 0.518 | | -0.057 (0.062) | 0.363 | 0.541 | 0.140 |
| *Solobacterium* | Genus | 0.096 (0.072) | 0.184 | 0.014 (0.025) | 0.582 | | 0.032 (0.038) | 0.402 | 0.146 | 0.279 |
| *Solobacterium moorei* | Species | 0.099 (0.074) | 0.182 | 0.011 (0.026) | 0.685 | | 0.032 (0.041) | 0.439 | 0.206 | 0.262 |
| *Spirochaetaceae* | Family | 0.109 (0.053) | **0.038** | -0.052 (0.024) | **0.029** | | 0.021 (0.077) | 0.782 | 0.871 | 0.005 |
| *Streptococcaceae* | Family | -0.062 (0.039) | 0.115 | 0.03 (0.021) | 0.153 | | -0.009 (0.043) | 0.828 | 0.765 | 0.039 |
| *Streptococcus* | Genus | -0.061 (0.041) | 0.138 | 0.056 (0.02) | **0.005** | | 0.003 (0.056) | 0.951 | 0.847 | 0.011 |
| *Streptococcus oralis subsp. dentisani clade 058* | Species | -0.035 (0.043) | 0.415 | 0.092 (0.019) | **0.000** | | 0.035 (0.061) | 0.565 | 0.864 | 0.007 |
| *Synergistaceae* | Family | 0.032 (0.231) | 0.891 | -0.088 (0.034) | **0.010** | | -0.084 (0.044) | 0.057 | 0.000 | 0.609 |
| *Treponema* | Genus | 0.109 (0.052) | **0.036** | -0.051 (0.024) | **0.034** | | 0.022 (0.076) | 0.774 | 0.872 | 0.005 |
| *Treponema socranskii* | Species | 0.027 (0.053) | 0.609 | -0.061 (0.026) | **0.018** | | -0.029 (0.042) | 0.481 | 0.553 | 0.135 |
| *Treponema_unclassified* | Species | 0.073 (0.251) | 0.771 | -0.118 (0.048) | **0.014** | | -0.101 (0.076) | 0.180 | 0.000 | 0.455 |
| *Veillonella_unclassified* | Species | -0.076 (0.178) | 0.668 | 0.046 (0.024) | 0.052 | | 0.039 (0.043) | 0.365 | 0.000 | 0.495 |
| *Veillonellaceae [G-1] bacterium HMT 155* | Species | -0.043 (0.073) | 0.553 | -0.034 (0.109) | 0.754 | | -0.041 (0.061) | 0.504 | 0.000 | 0.945 |


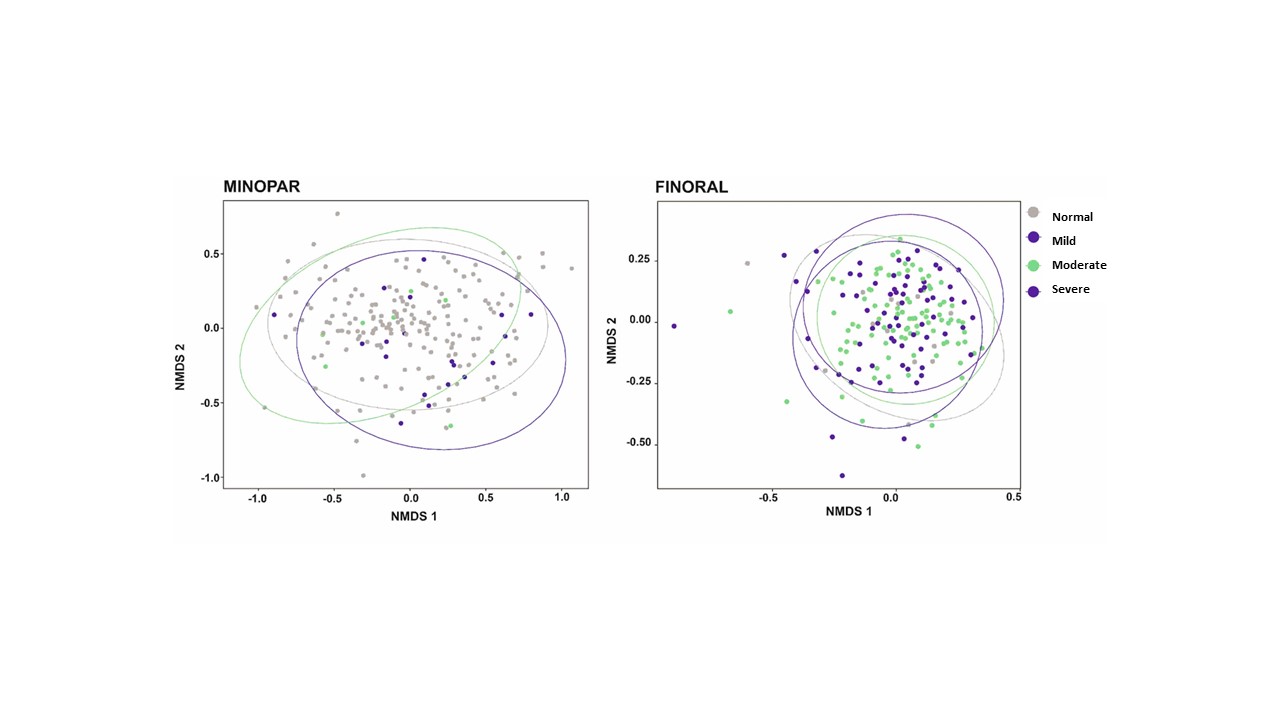


**Cohort 2**

**Cohort 1**

**Supplemental Figure 1.** Cohort 1 (n=202) and Cohort 2 (n=174) participants were divided into subgroups according to the MMSE score as normal, mild, moderate, and severe cognitive decline. Beta diversity was calculated for the subgingival microbiome composition in both cohorts. Non-metric Multidimensional Scaling (NMDS) plot visualization of beta diversity, using the Bray-Curtis distances matrix in Cohort 1 and Cohort 2**.**


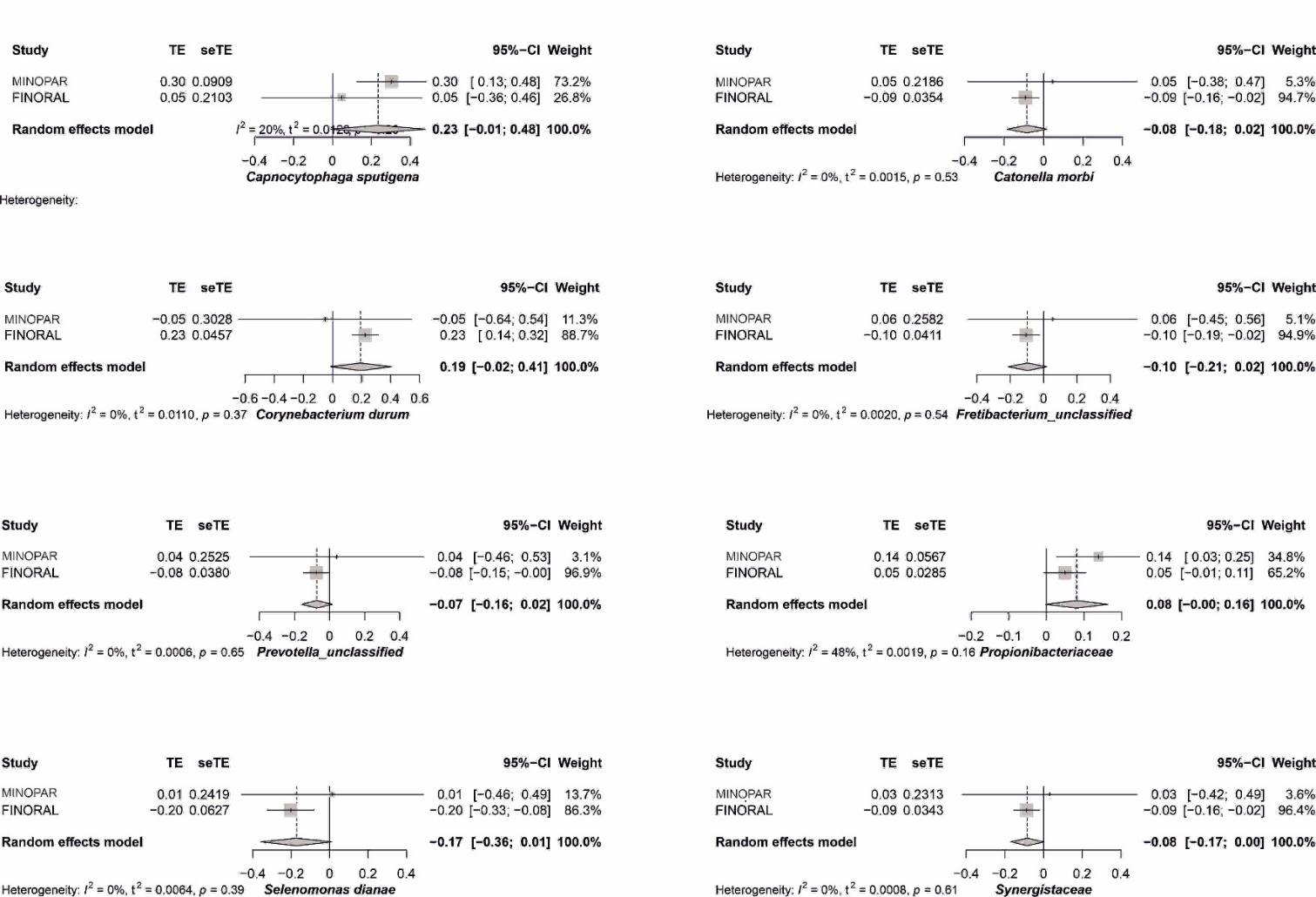


**Cohort 1**

**Cohort 2**

**Cohort 2**

**Cohort 1**

**Cohort 2**

**Cohort 2**

**Cohort 1**

**Cohort 2**

**Cohort 2**

**Cohort 1**

**Cohort 2**

**Cohort 2**

**Cohort 1**

**Cohort 2**

**Cohort 2**

**Cohort 1**

**Cohort 2**

**Cohort 2**

**Cohort 1**

**Cohort 2**

**Cohort 2**

**Cohort 1**

**Cohort 2**

**Cohort 2**

**Supplemental Figure 2. The forest plots of the taxa in the random effect models.** Cohort 1 (n=202) and Cohort 2 (n=174) had their subgingival microbiome composition determined and cognitive function investigated by MMSE. The forest plots display the association between the microbial taxa and MMSE (continuous variable) of the final random effects models. They were produced by meta-analyses of all significant taxa in either cohort, adjusted for age, sex, number of medications, PPD≥ 6 mm and presence of caries. Effect size and 95% CI are shown separately for Cohort 1, Cohort 2, and their meta-analyses.
