# Supplementary figures and images for: Subgingival microbiome at different levels of cognition
Source: J Oral Microbiol. 2023 Feb 19;15(1):2178765. doi: 10.1080/20002297.2023.2178765 (PMC9946326; doi:10.1080/20002297.2023.2178765)

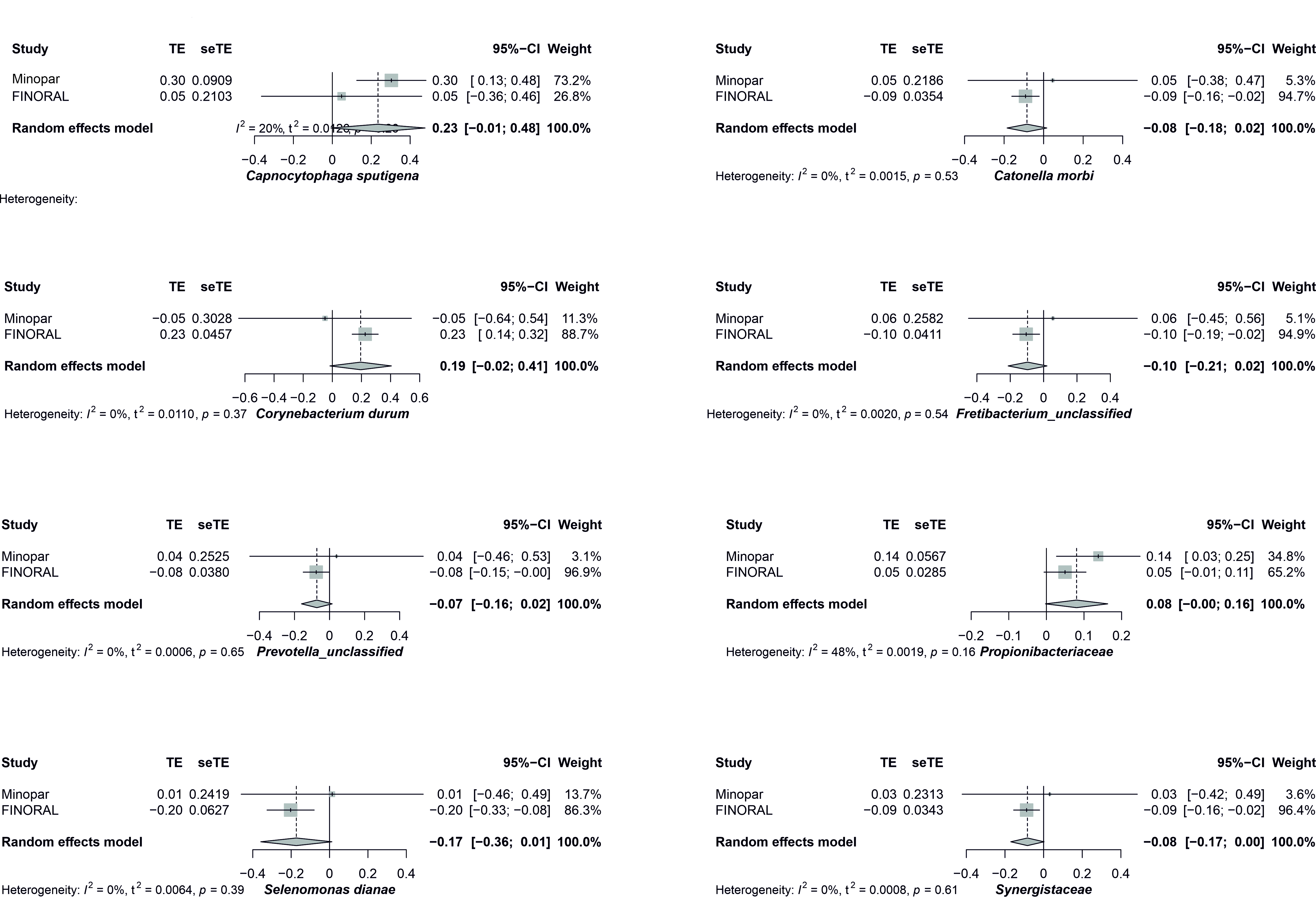

Supplement: Supplemental Material [file ZJOM_A_2178765_SM8440.zip › Supplementary files/Supplemental_Figure_2..jpg]
